# Supplementary figures and images for: The influence of neuromuscular blockade on phase lag entropy and bispectral index: A randomized, controlled trial
Source: PLoS One. 2021 Sep 14;16(9):e0257467. doi: 10.1371/journal.pone.0257467 (PMC8439464; doi:10.1371/journal.pone.0257467)

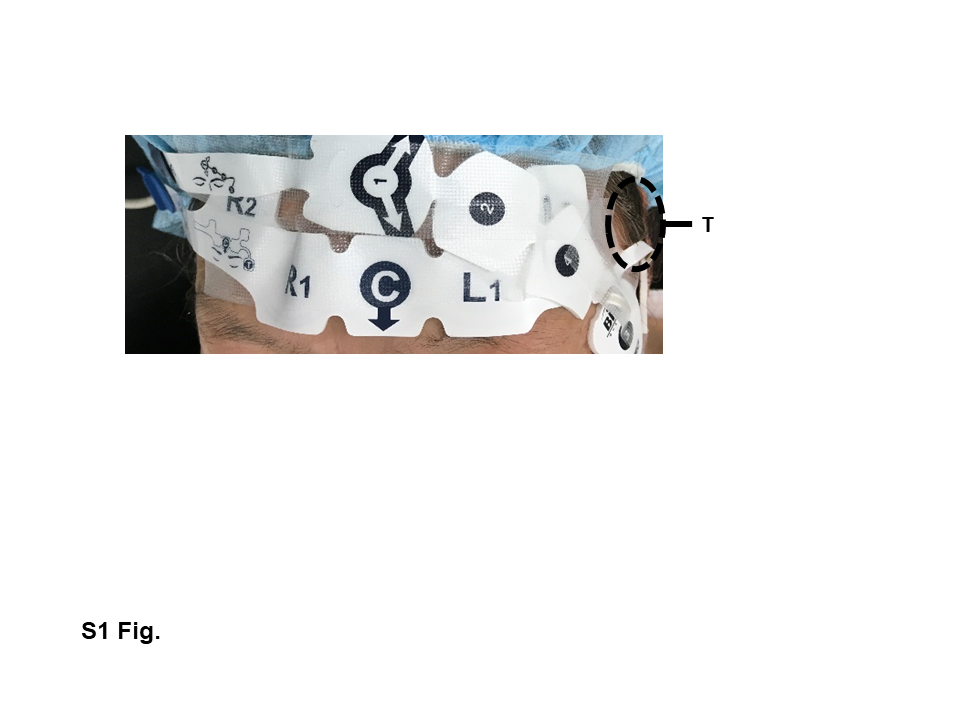

Supplement: S1 Fig — Upper BIS sensor; 1: reference electrode (FPz); 2: ground electrode (F3); 3: FT9; 4: measuring electromyography activity of the frontalis muscle (AF7). Lower PLE sensor; L1: Fp1, R1: Fp2, L2: AF3, R2: AF4, C: ground electrode, T: reference electrode. (TIF) [file pone.0257467.s002.tif]

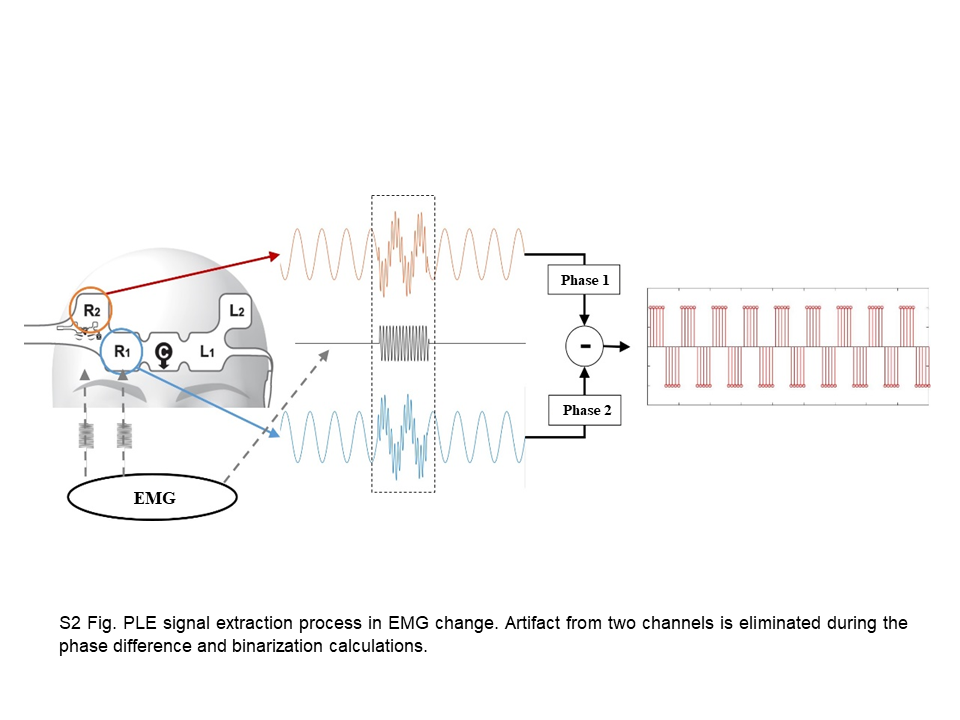

Supplement: S2 Fig — Artefacts from two channels were eliminated during the phase difference and binarization calculations. (TIF) [file pone.0257467.s003.tif]
