# Supplementary material for: The influence of neuromuscular blockade on phase lag entropy and bispectral index: A randomized, controlled trial
Source: PLoS One. 2021 Sep 14;16(9):e0257467. doi: 10.1371/journal.pone.0257467 (PMC8439464; doi:10.1371/journal.pone.0257467)
Supplement: S2 Table — (DOCX) [file pone.0257467.s005.docx]

**S2 Table. Demographic and Clinical Data in Patients under Sedation Based on Bispectral Index > 60 at before Administration of Study Drug**

|  | **Group C**  **(n = 9)** | **Group R**  **(n = 13)** |
| --- | --- | --- |
| Age (years) | 52.0 ± 7.8 | 48.1 ± 7.7 |
| Gender (Male : Female) | 8 : 1 | 7 : 6 |
| Weight (kg) | 73.1 ± 10.3 | 64.6 ± 11.1 |
| Height (cm) | 169.1 ± 7.5 | 166.8 ± 9.1 |
| Surgical department (N (%)) |  |  |
| OS | 10 (55.6) | 8 (42.1) |
| URO | 4 (22.2) | 5 (26.3) |
| GS | 3 (16.7) | 5 (26.3) |
| OBGY | 1 (5.6) | 1 (5.3) |

Values are mean ± SD or number (% of group). Group C: group who received same volume of normal saline as rocuronium, Group R: group who received rocuronium 0.6 mg/kg.
