# Supplementary material for: The influence of neuromuscular blockade on phase lag entropy and bispectral index: A randomized, controlled trial
Source: PLoS One. 2021 Sep 14;16(9):e0257467. doi: 10.1371/journal.pone.0257467 (PMC8439464; doi:10.1371/journal.pone.0257467)
Supplement: S3 Table — (DOCX) [file pone.0257467.s006.docx]

**S3 Table. Changes in Vital Signs after Administration of Study Drug at the End of Surgery**

|  | Group | TRV0 | TRV1 | TRV2 | TRV3 | TRV4 |
| --- | --- | --- | --- | --- | --- | --- |
| MBP  (mmHg) | D  (n = 19) | 80.4 ± 16.5 | 80.0 ± 14.2 | 80.9 ± 14.5 | 81.1 ± 15.6 | 82.3 ± 17.4 |
|  | B  (n = 18) | 80.9 ± 15.2 | 79.5 ± 12.7 | 77.9 ± 10.4 | 76.8 ± 10.4 | 76.8 ± 10.7 |
| HR  (/min) | D  (n = 19) | 64.5 ± 9.5 | 64.5 ± 9.1 | 64.8 ± 9.7 | 64.5 ± 9.2 | 64.6 ± 9.6 |
|  | B  (n = 18) | 60.4 ± 7.2 | 59.9 ± 6.7 | 59.6 ± 6.8 | 59.4 ± 7.0 | 60.2 ± 7.2 |

Values are mean ± SD. MBP: mean blood pressure, HR: heart rate, TRV0, TRV1, TRV2, TRV3, and TRV4: immediately before, 1, 2, 3, and 4 min after injection of rocuronium (group R) or same volume of normal saline as rocuronium (group C), respectively. There were no significant differences as time goes on and between the two groups.
