# Supplementary material for: The influence of neuromuscular blockade on phase lag entropy and bispectral index: A randomized, controlled trial
Source: PLoS One. 2021 Sep 14;16(9):e0257467. doi: 10.1371/journal.pone.0257467 (PMC8439464; doi:10.1371/journal.pone.0257467)
Supplement: S1 File — (DOCX) [file pone.0257467.s007.docx]

**Research Proposal**

1. **Title of the research**

The influence of neuromuscular blockade on phase lag entropy and bispectral index

1. **Name and address of the institution conducting the research**

Ewha Womans University Mokdong Hospital, Department of Anesthesia and Pain medicine

1071, Anyangcheon-ro, Yangcheon-gu, Seoul, Republic of Korea

1. **Research Director and manager**
2. **Research Director:** Professor Hee Jung Baik
3. **Co-researcher:** Not Applicable
4. **Research manager:** Resident So Hee Jin
5. **Pharmacists for clinical trials /** **Name and job name of the medical device manager for clinical trials:** Resident So Hee Jin
6. **Research request agency**
7. **Name of research request agency:** Not Applicable
8. **Monitor agent’s job name and name:** Not Applicable
9. **Sponsor organization**
10. **Organization name:** Inbody
11. **Diseases studied in the research**

Not applicable

1. **Background and goal of research**
2. **Background**

Various monitoring devices have been used to measure the depth of sedation and anesthesia in clinical practice. The bispectral index (BIS) is a processed electroencephalogram (EEG) parameter and a quantifiable measure of the depth of sedation and anesthesia. It is a single score on a scale from 0 to 100. It is known that neuromuscular blocking agents (NMBAs) affect the BIS. NMBAs reduce the BIS during sedation or light general anesthesia but does not affect it during general anesthesia1. Therefore, it is necessary to consider the effects of NMBAs when reading BIS levels during light sedation. Reversal of NMBAs changes the BIS in two ways. One is the interference effect on the BIS resulting from electromyograph (EMG) activation, and the other is that the muscle stretch receptor stimulates the arousal center of the brain (afferentation theory) ^[[1]](#footnote-1)^2.

The phase lag entropy (PLE) is a novel device and different from conventional devices, which do not provide information on communication between brain regions after analyzing single-channel EEG signals. The PLE extracts the phase relationship pattern from multichannel EEG signals of prefrontal and frontal cortex and calculates the entropy to predict the complexity of the communication between brain regions. PLE is calculated by combining PLE1, PLE2, and BSR (burst-suppression ratio) with appropriate weights. Although the frequency ranges of PLE2 are 0.1–1 Hz and 30–45 Hz, which include an EMG frequency band of ≥30 Hz, PLE does not use power spectrum analyses, unlike the BIS, which adopts the BetaRatio.

1. **Research hypothesis and goal**

Theoretically EMG changes caused by neuromuscular blockade do not affect PLE. However, there are no studies on this so far, so this study aims to compare the effect of neuromuscular blockade on PLE with BIS. We will compare the effects of NMBAs on PLE and BIS with a placebo after loss of consciousness during an induction of general anesthesia. In addition, at the end of the surgery, we will evaluate the effects of the reversal of NMBAs on PLE and BIS when the patients are still in deep sedation.

1. **Drug and medical device code name for clinical trials**

Medical equipment: Inbody Co., Ltd. EEG, PLEM100

(Medical device testing 10-03-20170014-0182 (2017.02.10), Korea Testing Certification Institute)

1. **Criteria for selection and exclusion, target number of subjects and basis for calculation**
2. **Selection criteria**

We will enroll the patients who are aged 19 to 60 years, American Society of Anesthesiologists physical status 1 or 2, and scheduled for various elective surgeries under general anesthesia.

1. **Exclusion criteria**

Patients are excluded if they had cardiopulmonary, hepatic, renal, neurological or neuromuscular disorders, or if they are pregnant, or taking sedatives, anti-depressants, or sleeping drugs.

1. **The target number of subjects and basis for calculation**

The sample size was calculated based on Cohen’s formula with two-sided testing using G*Power program version 3.1.9.2. According to the results that BIS value of the previous study3, the number of patients calculated with significant level (α error) of 0.05 and statistical power (1 - β error) of 0.8 was 16 per each group. Considering the dropout rate of 20%, the number of each group was 20.

1. **Recruitment plan**

For patients who are planning to perform elective surgery under general anesthesia through an outpatient clinic or hospitalization at the hospital, it is intended for patients who are planning to have surgery that can attach a PLE sensor to their forehead. After the end of operation, it is intended for patients who are expected to be transferred to the recovery room after neuromuscular blockade reversal and extubation of endotracheal tube. If patients meet the selection criteria of the study, every effort will be made to ensure that they participate in this study, and the goal of study will be noted to them so that they can represent the entire population of patients undergoing surgery at the institution.

1. **Estimated research period: IRB approval ~ May 31^th^ 2018**
2. **Research method**
3. **Specific research method**

After arrival in the operating room without premedication, noninvasive blood pressure, the electrocardiogram, and oxygen saturation (SpO_2_) are monitored.

A BIS sensor and a PLE sensor are attached to the patient’s forehead. After a 3–5 min stabilization period to achieve a signal quality index (SQI) of the BIS and PLE > 50, we record the baseline BIS, EMG on a BIS monitor, the baseline PLE, EMG on a PLE monitor in the eye-open state, BSR, BP, and HR.

The pharmacokinetic models of Schnider and Minto are used for propofol and remifentanil target-controlled infusion (TCI) through infusion pump (Orchestra^®^, Fresinius vial, France), respectively. After administering 0.2 mg glycopyrrolate, remifentanil TCI was started at a 2 ng/ml target effect-site concentration (Ce). After a stable remifentanil Ce was achieved, propofol TCI is started at a target Ce of 2 μg/ml, which will be increased by 0.5 μg/ml until loss of consciousness (LOC). LOC is defined as no response to verbal commands to open the eyes. When LOC is attained, the propofol Ce will be held constant during the study period at induction and the BIS, EMG_BIS, PLE, EMG_PLE, TOF count and ratio, blood pressure, and heart rate are recorded at 1-min intervals for 3 min. Then, 0.6 mg/kg rocuronium (group R) or the same volume of saline (group C) is injected intravenously. Assisted manual ventilation is performed with 100% oxygen via a face mask, and all measurements are recorded before and after administration of rocuronium at 1-min intervals for 4 min. Thereafter, the target Ce of remifentanil is increased to 4 ng/ml and the target Ce of propofol is increased to maintain a BIS of 40–55. In group C and R, 0.6 mg/kg rocuronium and the same volume of normal saline are administered, respectively; 1.5 min later intubation will be performed and anesthesia will be maintained. The preparing of the test drug (rocuronium or normal saline) will be performed by someone who will not take part in the study so that the anesthetists who inject the test drug and record the measurements will be blinded for the group assignment.

The level of NMB will be assessed by train-of-four (TOF) stimulation of the ulnar nerve and the degree of the response of adductor pollicis muscle.

At the end of the operation, while the constant Ce of propofol was maintained to keep the BIS at 40–55 with a Ce of remifentanil of 2 ng/ml, 2 mg/kg (group S) sugammadex (Bridion^®^, MSD, Seoul, Korea) or the same volume of saline (group D) is administered when three or more counts appeared on the TOF. We record all of the same measurements as induction before and after drug administration for 4 min at 1-min intervals. At the end of surgery, patients in groups D and S~~B~~ are given glycopyrrolate and pyridostigmine or the same volume of normal saline, respectively, and will be awakened from anesthesia by stopping the TCI.

First evaluation variable is the changes in BIS and PLE after administration of the neuromuscular blocking agent compared to the baseline during sedation. So ΔBIS and ΔPLE in group R are compared with them in the control group (group C) administered with normal saline. Second evaluation variable is the change in BIS and PLE before and after administration of reversal agent of the neuromuscular blockade, ΔBIS_R and ΔPLE_R. Therefore, the difference between the BIS and PLE with reversal agent of the neuromuscular blockade (the ΔBIS_R and ΔPLE_R) in group S~~B~~ is compared with them in group D administered with saline. In addition, the EMG changes on BIS and PLE before and after neuromuscular blocking agents and reversal agent of neuromuscular blockade are compared, and the difference in the subparameters of the BIS (SynchFastSlow, BetaRatio) and the PLE (PLE1, PLE2) will be calculated using the stored data.

1. **Control group and randomization method**

The experimental group and the control group are randomly assigned by a random number table made by a third party. Double-blind method is also used so that the anesthesiologist who give patients study drugs doesn’t know whether it is a placebo.

1. **Administration of test drug and control drug: the amount and the method**

For the propofol and remifentanil target-controlled infusion (TCI) through infusion pump (Orchestra^®^, Fresinius vial, France), pharmacokinetic models of Schnider and Minto are used, respectively. After administration of 0.2 mg of glycopyrrolate, remifentanil TCI was started at 2 ng/ml of target effect site concentration (Ce). After a stable remifentanil Ce is achieved, propofol TCI was started at target Ce of 2 μg/ml and it will be increased by 0.5 μg/ml until the loss of consciousness (LOC). LOC is defined as no response to verbal command to open the eyes. When LOC has been attained, propofol Ce is held constant during induction and BIS, EMG_BIS, PLE, EMG_PLE, train of four (TOF) count and ratio, blood pressure, and heart rate are recorded in 1-min intervals for 3 min. And then, rocuronium 0.6 mg/kg (group R) or same volume of saline as rocuronium (group C) is injected intravenously. Assisted manual ventilation is performed with 100% oxygen via facial mask and all measurements are recorded before and after administration of rocuronium in 1-minute intervals for 4 min. Thereafter, target Ce of remifentanil will be increased to 4 ng/ml and target Ce of propofol is increased to maintain the BIS 40-55. In group C and R, the rocuronium 0.6 mg/kg and same volume of normal saline is administered, respectively, and 1.5 min later, the intubation will be performed, and anesthesia will be maintained.

At the end of operation, while constant Ce of propofol is maintained to keep the BIS 40-55 with Ce of remifentanil of 2 ng/ml, sugammadex (Bridion^®^, MSD, Seoul, Korea) 2 mg/kg (group S~~B~~) or same volume of saline as sugammadex 2 mg/kg (group D) are administered when 3 or more counts appear on the TOF. We record all same measurements as induction before and after drug administration for 4 minutes in 1-minute intervals. Study drugs were prepared by a third party. After the end of the study process, patients in group D and S~~B~~ are given glycopyrrolate + pyridostigmine and same volume of normal saline as glycopyrrolate + pyridostigmine, respectively, and will be awakened from anesthesia by stopping the TCI.

1. **Observation items, Clinical laboratory tests, and the methods of them**

A BIS sensor and a PLE sensor are attached to the patient’s forehead. After a 3–5 min stabilization period to achieve a signal quality index (SQI) of the BIS and PLE > 50, we record the baseline BIS, EMG on a BIS monitor (EMG_BIS), PLE, and EMG on a PLE monitor (EMG_PLE). When LOC is attained after administration of anesthetics. all measurements are recorded before and after administration of rocuronium or saline in 1-minute intervals for 4 min. At the end of operation, we record all same measurements as induction before and after sugammadex or saline for 4 minutes in 1-minute intervals. SyncFastSlow, BetaRatio, PLE1, and PLE2 are calculated using the saved data.

1. **Points of difference from previous studies**

This is the first study that the effect of neuromuscular blockade on PLE is compared with BIS. If PLE, a developed anesthetic depth monitoring device, is not affected by EMG, it can replace BIS, which is known to be disturbed by EMG, so that the patient's sedation state can be more accurately evaluated regardless of the degree of neuromuscular blockade. That is, the use of PLE monitor could prevent awareness during anesthesia or overdose of anesthetics.

.

1. **Risk and benefit analysis of research subjects**

- The attached sensor can leave marks on subjects' foreheads, which will be noted to patients in advance. For BIS sensor currently widely used, there is a fine hook-shaped sucker in a sensor mounting portion, which can induce a mark after long time use. However, the risk is expected to be minor because the mark will disappear after several minutes or hours.
- Thorough monitoring of anesthesia depth reduces the risk of arousal during anesthesia.
- There is no cost burden for reversal agents of neuromuscular blockade according to group by randomization.

1. **Criteria for stopping research or dropping out**

When the patient is transferred to the intensive care unit without reversing the neuromuscular blockade due to an unexpected event by the changes in the patient's condition during surgery, or when anesthesia must be terminated in a sedative state.

1. **Safety evaluation criteria including side effects, evaluation methods and reporting methods**

If a side effect occurs after each patient is terminated, it is evaluated and a countermeasure is established and reported.

1. **Data Safety Monitoring Plan (DSMP)**

Not Applicable (The attached sensor is considered to be the minimum risk considering that BIS and PLE are widely used today and also have similar type of sensor.)

1. **Methods of data analysis and statistical analysis**

For all data management and statistical analyses we use SPSS version 20.0 (SPSS Inc, Chicago, Illinois, USA). An independent t-test or ANOVA are used to find out the difference between the control group and the experimental group. The p-value less than 0.05 is considered statistically significant.

1. **Research schedule**

-Recruitment: From the date of IRB approval to December 2017.

- Clinical research: Sort by creating a case of recording sheets anesthesia.

- Total research period: 12 months from the date of IRB approval

- June 2017-July 2017: IRB audit

- July-December 2017: Patient selection and data collection

- January-February 2018: Data collection and data organization

- March-May 2018: Final data collection and paper writing

1. **Safety plan for the protection of research subjects**
2. **Basic plan for securing research ethics**

This study will be conducted in accordance with the regulations of the World Medical Association Declaration of Helsinki's Ethical principles for medical research involving human subjects and ICH-GCP. The research process is carried out after deliberation and approval by the Institutional Review Board. The confidentiality of all information obtained from the clinical research study is maintained by the clinical research people and all others involved.

1. **Research subject consent process**
2. Researcher who will explain to research subjects and obtain consent: Research manager
3. Who will provide consent: Research subject or attorney
4. Waiting time between the process of explaining the study and obtaining consent: 30 minutes
5. How to minimize the likelihood of forced or undue influence: We have enough time for explanation and understanding.
6. Language used by researchers in the process of explaining research and obtaining consent: Korean or English
7. Language that the research subject or attorney can understand: Korean or English
8. Information provided to research subject or attorney: Research Manual 1 copy and Informed Consent 1 copy
9. **Plan for compensation for subject in the study**

There is no compensation provided to the study subjects, but sugammadex, which is used for reversal of neuromuscular blockade in the study, is a drug without benefit from health insurance and is expensive, so it is not borne by the study subjects and is paid from the research fund. There is no additional medical service fee incurred to the study subjects due to the research.

1. **Plan for personal information protection of subject in the study**

Only the research director and person in charge of this study have the relevant records. In order to prevent inadvertent exposure of information, a database for clinical trials provided by the Korean Society of Anesthesiology and Pharmacology is established by encoding the subject identification information in documents, and as an electronic document in a web-based case report form (web-CRF). After storage, we discard the paper document. All records are kept for 3 years from the end of the study, and matters related to personal information in the documents after their retention period will be destroyed in accordance with Article 16 of the Enforcement Decree of the Personal Information Protection Act.

1. **Additional protective plan in case of including vulnerable research subjects**

Not Applicable

1. **Methods for storage and disposal of human materials**

Not Applicable

1. **References**

Liu N et al. The influence of a muscle relaxant bolus on bispectral and Datex-Ohmeda entropy values during propofol-remifentanil induced loss of consciousness. Anesth Analg 2005; 101:1713-8

2 Lanier WL, Iaizzo PA, Milde JH, Sharbrough FW. The cerebral and systemic eﬀects of movement in response to a noxious stimulus in lightly anesthetized dogs. Possible modulation of cerebral function by muscle aﬀerents. Anesthesiology 1994; 80: 392-401.

3 Yun Chul Shin et al. The Influence of a Muscle Relaxant on Bispectral Index during the Propofol Induction of Anesthesia. Korean J Anesthesiol 2008; 54: 373-7.

1. 2 [↑](#footnote-ref-1)
