# Supplementary material for: The influence of neuromuscular blockade on phase lag entropy and bispectral index: A randomized, controlled trial
Source: PLoS One. 2021 Sep 14;16(9):e0257467. doi: 10.1371/journal.pone.0257467 (PMC8439464; doi:10.1371/journal.pone.0257467)
Supplement: S2 File — (DOC) [file pone.0257467.s008.doc]

**연구계획서(version:4.0)**

1. **연구의 명칭 및 단계**

신경근차단이 위상지연엔트로피와 바이스펙트럼지수에 미치는 영향

The influence of neuromuscular blockade on phase lag entropy and bispectral index

1. **연구의 실시기관명 및 주소**

이화여자대학교 의과대학부속 목동병원 마취통증의학과

서울시 양천구 안양천로 1071

1. **연구책임자 및 담당자 등**
2. **연구책임자:** 교수 백희정
3. **공동연구자:** 해당 없음
4. **연구담당자:** 전공의 진소희
5. **임상시험용 의약품 관리약사/임상시험용 의료기기 관리자 성명 및 직명:** 전공의 진소희
6. **연구 의뢰기관**
7. **연구 의뢰기관 명칭:** 해당 없음
8. **모니터요원 직명 및 성명:** 해당 없음
9. **연구비 지원기관**
10. **연구비 지원기관 명칭:** 인바디(Inbody)
11. **연구 대상 질환**
12. **연구의 배경 및 목적**
13. **연구배경**

진정이나 마취를 시행할 때 환자의 의식정도를 측정하는 다양한 종류의 감시장치가 개발되어 임상에서 사용되고 있는데, 바이스펙트럼지수(bispectral Index, BIS)는 뇌파(electroencephalogram, EEG)의 바이스펙트럼분석을 통해 마취제에 의한 진정 수면 효과를 0에서 100사이의 수치로 제공한다. 그러나 신경근차단제 투여는 BIS에 영향을 미치는 것으로 알려져 있으며, 많은 연구에서 얕은 진정 상태에서는 신경근차단제가 BIS를 감소시키는 반면, 깊은 마취 상태에서는 BIS에 영향을 주지 않았다[[1]](#endnote-2)고 보고하고 있어 특히 마취심도가 얕은 진정상태에서는 BIS 수치를 읽을 때 신경근차단 효과를 고려해야 한다. 신경근차단제 역전이 BIS의 변화를 일으키는 원인으로 두 가지를 들 수 있는데, 하나는 근전도(electromyography, EMG) 활성도에 의한 BIS 내 방해 효과이고, 다른 하나는 근육의 신장수용체(stretch receptor)가 뇌의 각성중추를 자극하여 각성효과를 나타낸다는 구심성 이론(afferentation theory)[[2]](#endnote-3)이다.

최근 새로 개발된 위상지연엔트로피(phase lag entropy, PLE)는, 단일채널 뇌파신호의 분석에 의존하여 뇌 영역간 커뮤니케이션 정보를 제공해 주지 못하는 기존의 장비들과는 달리, 전전두엽과 전두엽의 다채널 뇌파신호에서 위상관계 패턴을 추출한 후 정보 엔트로피를 계산하여 뇌 영역간 커뮤니케이션의 복잡성을 예측할 수 있는 새로운 개념의 진정, 마취 심도 장비이다. PLE는 세가지 하부변수, 즉, PLE1, PLE2, BSR(burst-suppression ratio)의 적절한 가중치를 두어 계산되는데 이 중에서 PLE2의 주파수대역이 0.1-1 Hz, 32-45 Hz로 30 Hz 이상의 EMG 주파수 대역을 포함하지만, 주파수 대역의 파워 값을 계산하는 베타 비(beta ratio)를 하부 변수로 채택하는 BIS와는 달리 파워 값을 계산하지 않는다.

1. **연구 가설 및 목적**

신경근차단에 의한 근전도 변화에 의해 위상지연엔트로피는(PLE)에 영향을 미치지 않을 것으로 생각된다. 그러나 아직까지 이에 대한 연구가 없으므로 본 연구에서는 신경근차단이 PLE에 미치는 영향을 BIS와 비교하고자 한다. 마취 유도 시 의식 소실 상태에서 신경근차단제 투여 후 BIS와 PLE의 변화를 측정하여 위약투여 군과 비교하며, 수술 종료 후 깊은 진정 상태에서 신경근차단 역전 시 변화가 있는지 비교하여 마취 심도 평가에 미치는 영향에 대해 평가하고자 한다.

1. **임상시험용 의약품 및 의료기기 코드명(또는 주성분의 일반명), 원료약품의 분량, 제형 등(대조약 포함)**

의료기기: (주)인바디. 뇌파계, PLEM100

(제 의기심 10-03-20170014-0182호 (2017.02.10) 한국기계전기전자시험연구원)

1. **연구대상자의 선정기준, 제외기준, 목표한 대상자 수 및 산출 근거**
2. **선정기준**

전신마취 하에 이루어지는 다양한 수술이 예정된 환자. 미국마취과학회 신체등급 분류 1, 2급에 해당되는 19-60세 환자 40명을 대상으로 한다.

1. **제외기준**

심장, 간, 신장, 중추 신경계 질환의 기왕력이 있는 환자나 진정제, 항우울제, 수면제를 복용하고 있는 환자, 임산부는 연구대상에서 제외한다.

1. **목표한 대상자 수 및 산출 근거**

G*Power 3.1.9.2 를 사용하여 양측검정으로 Cohen의 공식에 따라 바이스펙트럼지수를 관찰한 이전 연구[[3]](#endnote-4) 결과인 바이스펙트럼지수 변동치(신경근차단제 투여군: 12.9 ± 6.2, 대조군: 6.2 ± 6.7)를 사용하여, 유의수준(α error)는 0.05, 검정력(1- β error)은 0.8로 하여 계산한 표본 수는 각 군당 16명으로 산출되었다. 중도탈락율 20%를 고려하여 각군의 수는 20명으로 하였다.

1. **연구대상자 모집 계획**

본원 마취전진료 외래 방문 또는 입원을 통해 계획된 전신마취 하 수술을 시행할 예정인 환자를 대상으로 이마에 PLE 센서를 붙일 수 있는 수술이 계획된 환자를 대상으로 한다. 수술 종료 후 신경근차단 역전 후 발관하여 회복실 이송이 예상되는 환자를 대상으로 한다. 이 연구의 선정기준에 합당하다면, 가능한 환자들이 이 연구에 참여할 수 있도록 모든 노력을 다할 것이며 본 기관에서 수술을 받는 환자의 전체를 대표할 수 있도록 환자들에게 연구의 목적을 주지시킬 것이다.

1. **예상연구기간: IRB 승인일~2018년 5월 31일**
2. **연구방법**
3. **구체적인 연구방법**

전투약 없이 수술실에 도착 후 표준 감시인 심전도, 산소포화도, 비침습적 혈압을 측정한다. BIS(위쪽)와 PLE(아래쪽) 센서를 이마에 부착하여 SQI가 50이상이고, 수치가 안정화 기다린 후 3분 간 BIS, EMG on BIS, PLE, EMG on PLE, BSR, 혈압, 심박수를 기록한다.

마취유도는 Propofol-remifentanil 목표효과처농도 조절 주입으로(Orchestra^Ⓡ^, Fresenius vial, France), Minto와 Schneider 약동학적 모형을 사용한다. Glycopyrrolate 0.02mg/kg 투여 후 Remifentanil의 효과처농도가 2 ng/ml로 시작한다. Propofol의 목표효과처농도를 2㎍/ml로 시작하여 의식이 소실 될 때까지 0.5㎍/ml씩 증량한다. 의식 소실은 눈을 떠보라는 구두명령에 반응이 없는 경우로 한다. 의식 소실이 일어난 때의 propofol 효과처농도를 3분 간 유지하면서 1분 간격으로 BIS, EMG on BIS, PLE, EMG on PLE, BSR(Burst suppression ratio), 사연속자극(train of four, TOF), 혈압, 심박수를 측정한 후 rocuronium 0.6 mg/kg(R군) 또는 동량의 생리식염수(C군)를 정주한다. 필요한 경우 100% 산소로 보조 환기를 시행하며 신경근차단제 투여 4분 후까지 1분 간격으로 모든 측정치를 기록한다. 이후 remifentanil의 목표효과처농도를 4 ng/ml로 증가시키고 BIS 40-55를 유지하도록 propofol의 목표효과처농도를 증가시킨 후 R군은 동량의 생리식염수를, C군은 rocuronium 0.6mg/kg을 투여하고 1분 30초 후 기관내삽관을 하고 수술을 위한 통상적인 마취유지 방법을 제공한다. 투약을 실시하는 사람과 측정치를 기록하는 사람은 어떤 군에 환자가 속해 있는지 알 수 없도록 투약준비는 제3자에 의해 이루어진다.

의식 소실이 일어난 후부터 TOF를 측정하기 시작하고 신경자극기를 이용하여 손목의 척골신경에 사연속자극을 20초 간격으로 가하여 모지 내전근(adductor pollicis m.)의 근수축 반응으로 측정한다.

수술이 종료된 후, 신경근차단을 역전시키는 단계에서 깊은 최면 상태, 즉 BIS 40 ~ 55 미만을 유지하면서(이 때 remifentanil의 효과처농도는 2 ng/ml로 유지함) TOF count상 3개 이상의 반응이 나타날 때 sugammadex 2mg/kg(S군)나 이와 동량의 생리식염수(D군)를 투여하고, 약물 투여전과 투여 후 4분까지 1분 간격으로 모든 측정치를 기록한다. 이로써 연구는 종료하고 마취제 주입을 종료한 후 마취에서 각성시킨다. 단, 생리식염수를 투여한 군은 신경근차단 역전을 위해 통상적으로 사용하는 glycopyrrolate과 pyridostigmine을 투여한다.

1차 평가 변수는 얕은 진정 하 신경근차단제 투여 전에 비해 신경근차단제 투여 후의 BIS와 PLE의 변화치, 즉 ΔBIS와 ΔPLE로 생리식염수를 투여한 대조군의 ΔBIS와 ΔPLE와 각각 비교하고, 2차 평가 변수는 깊은 최면 하에서 신경근차단제 역전에 의해 신경근차단제의 효과가 사라졌을 때 신경근차단 상태에 비해 BIS와 PLE의 변화치, 즉 ΔBIS_R와 ΔPLE_R로, 생리식염수를 투여한 대조군의 ΔBIS_R와 ΔPLE_R과 각각 비교한다. 또한 이러한 변화에 영향을 미칠 수 있는 변수로서, 신경근차단제 및 신경근차단역전제 투여 전과 후의 BIS와 PLE 상의 EMG 변화치를 비교하고, 저장된 데이터를 이용하여 계산한 BIS의 하부모수(SynchFastSlow, BetaRatio)와 PLE의 하부모수(PLE1, PLE2)의 변화치 또한 비교한다.

1. **비교군 설정 및 무작위 배정 방법**

환자군과 대조군 배정을 하는 사람이 아닌 제3자에 의해 만들어진 난수표에 의해 무작위 배정되어 두군으로 나뉠 것이며 투약하는 사람 또한 위약인지 여부를 알 수 없는 이중맹검법을 사용할 것이다.

1. **시험약 투여∙사용량, 투여∙사용 방법, 병용 요법, 대조약 사용시 그 선택사유**

마취유도는 Propofol-remifentanil 목표효과처농도 조절 주입으로(Orchestra^Ⓡ^, Fresenius vial, France), Minto와 Schneider 약동학적 모형을 사용한다. Glycopyrrolate 0.02mg/kg 투여 후 Remifentanil의 효과처농도 2 ng/ml로 시작한다. Propofol의 목표효과처농도를 2㎍/ml로 시작하여 의식이 소실 될 때까지 0.5㎍/ml씩 증량한다. 의식 소실은 눈을 떠보라는 구두명령에 반응이 없는 경우로 한다. 의식 소실이 일어난 때의 propofol 효과처농도를 3분 간 유지하면서 1분 간격으로 BIS, EMG on BIS, PLE, EMG on PLE, BSR(Burst suppression ratio), 사연속자극(train of four, TOF), 혈압, 심박수를 측정한 후 rocuronium 0.6 mg/kg(R군) 또는 동량의 생리식염수(C군)를 정주한다. 필요한 경우 100% 산소로 보조 환기를 시행하며 신경근차단제 투여 4분 후까지 1분 간격으로 모든 측정치를 기록한다. 이후 remifentanil의 목표효과처농도를 4 ng/ml로 증가시키고 BIS 40-55를 유지하도록 propofol의 목표효과처농도를 증가시킨 후 R군은 동량의 생리식염수를, C군은 rocuronium 0.6mg/kg을 투여하고 1분 30초 후 기관내삽관을 하고 수술을 위한 통상적인 마취유지 방법을 제공한다. 투약을 실시하는 사람과 측정치를 기록하는 사람은 어떤 군에 환자가 속해 있는지 알 수 없도록 투약준비는 제3자에 의해 이루어진다.

수술이 종료된 후, 신경근차단을 역전시키는 단계에서 깊은 최면 상태, 즉 BIS 40 ~ 55 미만을 유지하면서(이 때 remifentanil의 효과처농도는 2 ng/ml로 유지함) TOF count상 3개 이상의 반응이 나타날 때 sugammadex 2mg/kg(S군)나 이와 동량의 생리식염수(D군)를 투여하고, 약물 투여전과 투여 후 4분까지 1분 간격으로 모든 측정치를 기록한다. 이로써 연구는 종료하고 마취제 주입을 종료한 후 마취에서 각성시킨다. 단, 생리식염수를 투여한 군은 신경근차단 역전을 위해 통상적으로 사용하는 glycopyrrolate과 pyridostigmine을 투여한다.

1. **관찰항목 및 임상검사항목 및 관찰검사방법**

BIS, PLE 센서를 이마에 부착하여 SQI가 50 이상이고, 수치가 안정화 될 때까지 약 3-5분 기다린 후, BIS, EMG activity on BIS, PLE, PLE on EMG, BSR, 혈압, 심박수 초기값을 기록한다. 이후 약물을 투여하면서 환자의 의식소실 여부와 함께 위의 값들을 신경근차단제 또는 생리식염수를 투여하고 4분 후까지 1분 간격으로 기록한다. 수술 중에는 TOF count 및 ratio를 기록한다. 수술 종료 후 sugammadex 또는 생리식염수를 투약한 후 4분까지 1분간격으로 위의 값들을 기록한다. 저장된 데이터를 이용하여 계산된 SyncFastSlow, BetaRatio, PLE1, PLE2를 기록한다.

1. **기존 치료 및 연구와의 차별점**

본 연구에서는 신경근차단이 PLE에 미치는 영향을 BIS와 비교하고자 한다. 새로 개발된 마취심도 감시장비인 PLE가 근전도로 인해 영향을 받지 않는다면, 근전도에 방해를 받는다고 알려진 BIS를 대체할 수 있어 신경근차단정도와 무관하게 환자의 진정 상태를 보다 정확하게 평가하여 실제 마취 중 각성이 일어나는 것을 줄일 수 있을 뿐 아니라, 근전도 활성에 의한 BIS 증가로 야기될 수 있는 약물의 과용을 줄일 수 있다.

1. **연구대상자의 위험/이익 분석**

연구대상자들의 이마에 부착되는 센서로 인한 자국이 남을 수 있다. 이에 대한 사실을 미리 고지하고 현재 널리 사용되고 있는 BIS 센서의 경우 센서 부착부위가 미세한 갈고리 모양의 흡착판으로 되어있어 장시간 부착 후 제거 시 생길 수 있는 자국은 수분내지 수시간 후 사라지므로 이에 대한 위험도는 크지 않을 것으로 사료된다.

마취 심도 감시를 철저히 함으로써 마취 중 각성에 대한 위험도가 줄어든다.

무작위 배정에 의해 군에 따라 신경근차단 역전 약에 대한 비용 부담이 없다.

1. **중지∙탈락 기준**

수술 중 예상치 못한 환자상태 변화로 환자를 신경근차단을 역전시키지 않고 중환자실로 이송하게 되거나 진정상태로 마취를 종료해야 하는 경우

1. **부작용을 포함한 안전성의 평가기준, 평가 방법 및 보고방법**

매 환자 종료 후 부작용이 발생한 경우 이에 대한 평가가 이루어지며 이에 대한 대책을 수립하고 보고한다.

1. **자료안전성 모니터링 계획(DSMP)**

해당 없음. (부착하는 센서는 현재도 널리 사용되는 BIS와 PLE 또한 이와 유사한 형태를 가진 센서이므로 최소위험에 해당한다고 생각된다.)

1. **자료 분석 및 통계 분석 방법**

자료 관리와 모든 통계 분석은 SPSS version 20.0 (SPSS Inc, Chicago, Illinois, USA)을 이용한다. 독립표본 T 검정을 시행하여 대조군과 실험군 간에 얕은 진정과 깊은 진정 시 어떠한 차이가 있는지 알아본다. 모든 검정은 p값이 0.05미만인 경우를 통계적으로 의미 있는 것으로 판정한다.

1. **연구수행일정표**

-대상모집: IRB 승인일로부터 2017년 12월까지 환자를 모집한다.

-임상연구: 마취 중 증례기록지를 작성하여 정리한다.

- 총 연구기간: IRB 승인일로부터~ 12개월

- 2017년 6월 ~ 2017년 7월: IRB 심사

- 2017년 7월 ~ 12월 : 대상 선정 및 자료 수집

- 2018년 1월 ~ 2월 : 자료 취합 및 데이터 정리

- 2018년 3월 ~ 5월: 최종 자료 취합 및 논문작성

1. **연구대상자의 안전보호를 위한 대책**
2. **연구의 윤리성 확보를 위한 기본 방안**

본 연구는 World Medical Association Declaration of Helsinki의 Ethical principles for medical research involving human subjects의 제반 규정 및 ICH-GCP를 준수하여 수행될 것이며, 연구의 제반 과정은 Institutional Review Board의 심의 및 승인을 취득 후 수행된다. 임상 연구로부터 얻은 모든 정보에 대해 임상연구 담당자나 그 외 관련된 모든 사람은 기밀성을 지킨다.

1. **연구대상자의 동의 과정**
2. 연구대상자에게 설명하고 동의를 취득할 연구자: 연구 담당자
3. 동의를 제공할 자: 연구대상자 또는 대리인
4. 연구 설명 과정과 동의 취득 과정 사이의 대기 시간: 30분
5. 강제 또는 부당한 영향의 가능성을 최소화시킬 방법: 충분한 설명과 이해의 시간을 가진다.
6. 연구 설명 과정과 동의 취득 과정에서 연구자가 사용하는 언어: 한국어 또는 영어
7. 연구대상자 또는 대리인이 이해할 수 있는 언어: 한국어 또는 영어
8. 연구대상자 또는 대리인에게 제공되는 정보: 연구설명서 및 동의서 1부
9. **연구대상자의 보상 방안**

연구대상자에게 제공되는 보상금은 없으나 연구에서 신경근차단 역전에 사용되는 sugammadex는 비급여약제로 고가이므로 연구대상자에게 부담시키지 않고 연구비에서 지급하며, 연구로 인해 연구대상자에게 추가로 발생하는 진료비는 없다.

1. **연구대상자의 개인정보보호 방안**

본 연구의 연구책임자와 담당자만이 해당 기록을 가지고 있으며 부주의한 정보노출을 방지하지 위해 문서에 대상자 식별정보를 코드화 및 암호화하여 대한마취약리학회에서 제공하는 임상시험용 데이터베이스를 구축하여 웹 기반 증례기록서(web-based case report form, web-CRF)에 전자문서로 저장한 후 종이 문서를 폐기한다.

모든 기록은 연구 종료시점부터 3년간 보관하며, 보관 기간이 지난 문서 중 개인정보에 관한 사항은 개인정보보호법 시행령 제 16조에 따라 파기될 것이다.

1. **취약한 연구대상자를 포함하는 경우 추가적인 보호조치 방안**

해당 없음

1. **인체유래물 연구의 경우 보관 및 폐기방법**

해당 없음

**14. 참고 문헌**

1. Liu N et al. The influence of a muscle relaxant bolus on bispectral and Datex-Ohmeda entropy values during propofol-remifentanil induced loss of consciousness. Anesth Analg 2005; 101:1713-8 [↑](#endnote-ref-2)
2. Lanier WL, Iaizzo PA, Milde JH, Sharbrough FW. The cerebral and systemic eﬀects of movement in response to a noxious stimulus in lightly anesthetized dogs. Possible modulation of cerebral function by muscle aﬀerents. Anesthesiology 1994; 80: 392-401. [↑](#endnote-ref-3)
3. Yun Chul Shin et al. The Influence of a Muscle Relaxant on Bispectral Index during the Propofol Induction of Anesthesia. Korean J Anesthesiol 2008; 54: 373∼7 [↑](#endnote-ref-4)
